# Open questions in boron species with globally 4*n* π systems

**DOI:** 10.1038/s42004-021-00542-x

**Published:** 2021-07-13

**Authors:** Kei Ota, Rei Kinjo

**Affiliations:** Division of Chemistry and Biological Chemistry, School of Physical and Mathematical Sciences, Nanyang Technological University, Singapore, Singapore

**Keywords:** Chemical bonding, Structure prediction

## Abstract

The Hückel rule defines that monocyclic and planar conjugated systems containing [4*n* + 2] π electrons are aromatic. Here, the authors highlight boron species that feature a globally 4*n* π system, defying the Hückel rule, but nonetheless exhibit aromaticity.

In 1931, Hückel introduced the electron counting protocol to account for the peculiar thermodynamic stability of the family of cyclic and planar hydrocarbons featuring delocalized [4*n* + 2] π-electron systems—now called Hückel aromaticity^1^. Later, Breslow proposed that cyclic molecules with 4*n* π electrons are antiaromatic^2^. Notably, the relationship between the [4*n* + 2]/4*n*-electron and its aromatic/antiaromatic nature can invert depending on the spin-state. That is, conjugated 4*n*-electron molecules in the lowest triplet state (T_1_) or the singlet excited state (S_1_) exhibit aromatic properties—Baird aromaticity^3^.

Nowadays, the concept of the aromaticity/antiaromaticity based on the [4*n* + 2]/4*n*-electron counting rule as well as the spin-state is not confined to organic compounds but is widely applied to various molecular systems ranging from organometallics to inorganic species. Moreover, in addition to archetypal π aromatics, other types such as σ-, δ-, and φ-aromatics are proposed, which has expanded the implication of the concept of aromaticity^4^. Irrespective of the system, the classical [4*n* + 2]/4*n*-electron counting approach still remains the standard method for the initial instant evaluation of the aromatic nature of molecules. In this context, are all planar compounds with 4*n* π systems in the singlet ground state always supposed to be anti-aromatic? In other words, can aromatic singlet molecules with a globally 4*n* π system exist?

## Is it possible to split a 4*n* π system into sub [4*n* + 2] π systems?

**Fig. 1:**
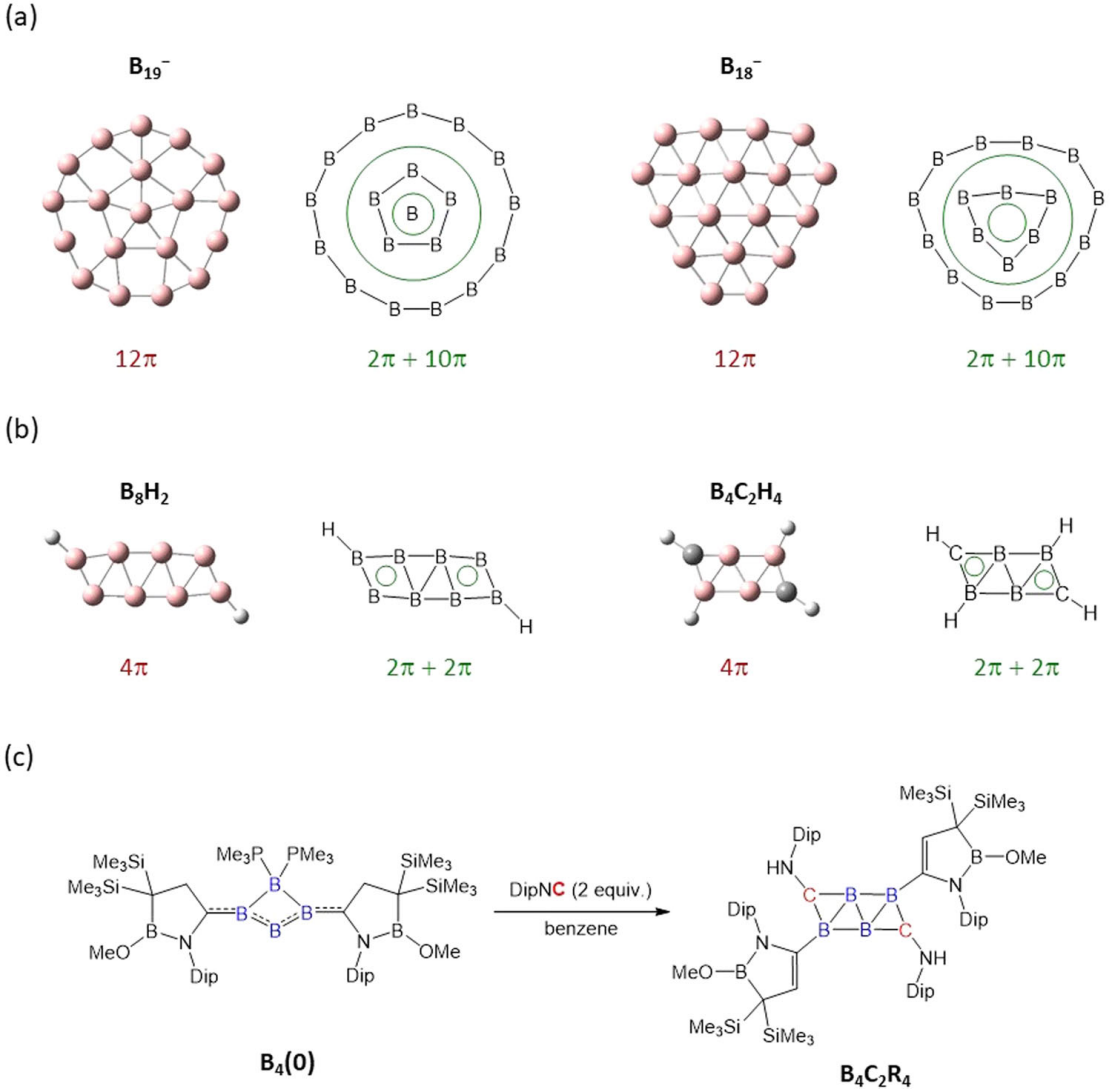
Aromatic boron species with globally 4*n* π electrons. **a** Molecular structures and schematic drawings of concentric doubly π-aromatic systems. **b** Molecular structures and schematic drawings of ribbon aromatic systems. **c** Synthesis of isolable B_4_C_2_ carborane (Dip = 2,6-diisopropylphenyl. Me = methyl).

In 2010, Wang, Boldyrev, and colleagues reported the **B**_**19**_^**–**^ cluster, which was generated by a laser vaporization method and detected by photoelectron spectroscopy (PES)^5^. Computational studies revealed that the global minimum (*C*_2v_) of **B**_**19**_^**–**^ exhibits a planar geometry with one central boron atom surrounded by a pentagonal B_5_ ring in the first coordination sphere and a B_13_ ring in the second coordination sphere (Fig. 1a). **B**_**19**_^**–**^ possesses in total 12 π electrons, suggesting that it is antiaromatic according to the [4*n* + 2]/4*n*-electron counting criteria. Nevertheless, its π aromatic nature is confirmed by the nucleus-independent chemical shift (NICS_zz_) value of −14.9 ppm at 0.6 Å above the **B**_**19**_^**–**^ plane as well as molecular orbital analysis. This can be rationalized by considering it as a concentric, doubly π aromatic system, in which two π electrons are delocalized over the central pentagonal B_5_ unit, and ten π electrons are delocalized in a circle between the central B_6_ fragment and the peripheral boron atoms, respectively. Consequently, each π-electron circulation simultaneously obeys the [4*n* + 2] Hückel rule—an example of concentric aromaticity. Interestingly, Heine, Merino, and colleagues revealed the fluxional behaviour of **B**_**19**_^**–**^. That is, the internal and external boron rings rotate nearly freely with respect to each other, like a Wankel motor^6^.

In 2011, the same group generated a **B**_**18**_^**–**^ species by the method described above and analysed it with PES^7^. According to ab initio calculations, one of the global minimum structures of **B**_**18**_^**–**^ (*C*_3v_), which is only slightly more stable by 1.6 kcal mol^−1^ than the other isomer (*C*_s_), is quasi-planar and consists of an inner triangular B_6_ unit and a peripheral B_12_ ring (Fig. 1a). **B**_**18**_^**–**^ involves the 6c–2e π bond delocalized over the inner B_6_ moiety and five π bonds (three 5c–2e and two 18c–2e) delocalized between the inner six B atoms and the peripheral ring. Accordingly, analogous to **B**_**19**_^**–**^, **B**_**18**_^**–**^ is also concentric doubly π aromatic. These studies illustrate that boron clusters even with globally 4*n* π electrons in the singlet state may in fact be aromatic by splitting them into several cyclically delocalized [4*n* + 2] π-electron systems. A subsequently emerging question is whether this way of dividing π electrons is limited to concentric circles.

## Can we cut 4*n* π systems into small strips?

In 2012, the group of Wang and colleagues reported a series of dehydrogenated boron clusters **H**_**2**_**B**_***x***_^**−**^ (*x* = 7–12) that were characterized by PES and computational studies^8^. The global minima of the clusters **H**_**2**_**B**_***x***_^**−**^ have ladder-like double-chain structures capped by a hydrogen atom at each terminal boron. The chemical bonding analysis revealed that the π bonding patterns in this class of the clusters bear an analogy to polyenes. Among them, the clusters **H**_**2**_**B**_**7**_^**−**^, **H**_**2**_**B**_**8**_, and **H**_**2**_**B**_**9**_^**−**^ are found to possess a 4π-electron system, indicative of antiaromatic character. Shortly thereafter, Li and colleagues reported that the B_4_ rhombus in the planar clusters of **H**_**2**_**B**_**4**_ (*D*_2h_), **H**_**2**_**B**_**8**_ (*C*_2h_), and **H**_**2**_**B**_**12**_ (*C*_2h_), is not only equivalent to a C=C double bond unit in the corresponding C_*n*_H_*n*+2_ (*n* = 2, 4, 6) hydrocarbons but possesses aromatic character (Fig. 1b)^9^. For instance, each terminal B_4_ rhombus in **H**_**2**_**B**_**8**_ is covered by a delocalized 4c–2e π bond and the NICS(0)_πzz_ is –22.9 ppm. Thus, breaking the 4π system into two 2π strips may equip the cluster with an aromatic feature—called ribbon aromaticity.

The aforementioned systems (**B**_**19**_^**–**^, **B**_**19**_^**–**^, **H**_**2**_**B**_**8**_) are transient species generated in situ and characterized only by PES. Is the chemical synthesis of aromatic 4*n* π electron systems possible?

## How to isolate and structurally characterize such species

Recently, our group developed a carborane **B**_**4**_**C**_**2**_**R**_**4**_ by the reaction of tetraatomic boron zero species **B**_**4**_**(0)** with isonitriles (Fig. 1c)^10,11^. **B**_**4**_**C**_**2**_**R**_**4**_ was obtained as orange crystals in 23% isolated yields. The solid-state molecular structure determined by an X-ray diffractometry shows that the skeletal B_4_C_2_ framework is perfectly planar with a double-chain structure, similar to **H**_**2**_**B**_***x***_^**−/0**^. Computational investigations show that the B_4_C_2_ core of **B**_**4**_**C**_**2**_**R**_**4**_ involves 4π electrons, as well as, the NICS value at 1.0 Å above the CBB three-membered ring is −11.8 ppm, in line with the NICS value of −15.0 ppm of the parent derivative **B**_**4**_**C**_**2**_**H**_**4**_. This indicates that the B_4_C_2_ core bears two delocalized 3c–2e π bonds (Fig. 1b), leading to the ribbon aromatics. In **B**_**4**_**C**_**2**_**R**_**4**_, (**H**_**2**_**B**_**8**_ as well) the central B_4_ rhombus has a 4c–2e σ-bond, forming the consecutive π- and σ-delocalized system^8,10^. In 2015, the group of Lu and Li proposed that such a unique conjugation system is expected to appear in longer double-chains as well^12^. Our work demonstrates that the synthesis of bottleable ribbon aromatic species is feasible by employing the proper substituents and synthetic approach.

## Outlook

Mathematically, the union of the numbers represented by [4*n* + 2] gives 4*m* (*m* = 2*n* + 1). Accordingly, as far as the construction of [4*n* + 2] π sub-units is plausible, even compounds with 4*n* π could have a chance to be aromatic, indicating that an instant judgement of aromaticity of molecules only by the Hückel rule needs to be avoided. The open questions remaining are as follows: Apart from concentric and ribbon aromatics highlighted above, what would other systems exist? What about other *p*-block elements-containing systems? Could we establish the protocol to predict the local aromaticity of 4*n* π molecules from their structure?

Both theoretical and experimental elaboration will lead to the answers to those questions. Computationally, further studies to explore molecules bearing the globally 4*n* and locally delocalized [4*n* + 2]-electron systems can be anticipated. Synthetically, isolation of such species remains highly challenging but we believe that it is attainable to develop them by the proper approaches. For instance, by employing the judiciously selected Lewis bases as the supporting ligands, an isolable version of the concentric aromatic species and more can be prepared. Not only are these species fundamentally significant synthetic targets, but their peculiar electronic structures should contribute to the development of nanomaterials.
